# Correlation between expression of Ki-67 and MSCT signs in different types of lung adenocarcinoma

**DOI:** 10.1097/MD.0000000000018678

**Published:** 2020-01-10

**Authors:** Jingang Yan, Heping Wang, Haiwei Zhou, Hui He, Lin Qiu, Zhaoyu Wang

**Affiliations:** a Department of Radiology; b Department of Pathology; c Medical Record Statistics Room, Zhoushan Hospital of Zhoushan City, Zhoushan, Zhejiang, PR China.

**Keywords:** immunohistochemistry, Ki-67, lung cancer, tomography, X-ray computed

## Abstract

To investigate the correlation between the proliferating cell nuclear antigen Ki-67 and the multislice computed tomography (MSCT) signs in different subtypes of lung adenocarcinoma.

Ninety-five patients with lung adenocarcinoma confirmed by surgical pathology and treated between January 2017 and December 2017 were included. MSCT was performed before the operation, and the characteristics of the high-resolution CT (HRCT) signs of the lesions were compared with the Ki-67 immunohistochemistry results.

The levels of Ki-67 in the 95 lung adenocarcinoma specimens were positively correlated with the malignancy of lung adenocarcinoma. Spearman correlation coefficient was 0.615. The expression of Ki-67 was positively correlated with the nodules’ diameter, density, and lobulated sign, with Spearman correlation coefficients of 0.58, 0.554, and 0.436. There was no significant correlation with spiculation and pleural retraction, with correlation coefficients of 0.319/0.381.

These findings suggest that the MSCT signs of different types of lung adenocarcinoma might be associated with the expression of Ki-67. Without replacing biopsy, the imaging features of pulmonary nodules could be comprehensively analyzed to evaluate the proliferation potential of preoperative nodules, but additional studies are needed for confirmation.

## 1 Introduction

Lung cancer is the leading cause of cancer-related deaths worldwide despite several advances in diagnosis and treatment, and its incidence has been steadily increasing in industrialized countries.^[1]^ Lung adenocarcinoma is the most commonly diagnosed histological subtype of non-small-cell lung cancer (NSCLC).^[2]^ In 2011, a new classification system for lung adenocarcinoma according to the International Association for the study of Lung Cancer (IASLC), American Thoracic Society (ATS), and European Respiratory Society (ERS) has been put forward, wherein the lung adenocarcinomas are mainly classified as adenocarcinoma in situ (AIS), minimally invasive adenocarcinoma (MIA), and invasive adenocarcinoma (IAC).^[3]^ Understanding the biological characteristics of this devastating disease could facilitate early diagnosis and individualized treatment, improving the survival rate.

Uncontrolled cell proliferation is considered as the key characteristic of cancer. Ki-67, a nuclear protein, is expressed during the active phases of the cell cycle, except in the G0 stage, and its proliferation index (PI) has been widely used as a marker of cell proliferation. According to previous studies, a high Ki-67 PI is associated with a negative impact on disease-free survival, relapse-free survival, and overall survival in patients with NSCLC.^[4,5]^ Hence, Ki-67 PI might be a valuable biomarker for predicting the prognosis of lung cancer patients. The conventional method for detecting Ki-67 expression is immunohistochemistry, which requires specimens obtained by invasive methods such as biopsy or surgery. Because of intratumoral heterogeneity, biopsy may miss the more aggressive foci within the tumor, leading to underestimation of the disease. Therefore, an accurate and noninvasive way of predicting the Ki-67 status in patients with lung cancer is clinically desirable.

Computed tomography (CT) is currently considered as the primary means for screening and monitoring lung cancer. Although some studies have evaluated the association of different pathological types with the Ki-67 index in lung tumors, there are no studies that investigated the relationship between the expression of Ki-67 and different subtypes of lung adenocarcinoma.

Hence, in this study, we primarily aimed to examine the correlation between CT features and Ki-67 status in lung adenocarcinoma. We also assessed whether the CT features could serve as noninvasive predictors of the Ki-67 status in patients with lung adenocarcinoma.

## 2 Methods

### 2.1 Study population

This retrospective study was approved by our institutional review board (Zhoushan Hospital of Zhoushan City, China). Medical review was performed in accordance with the institutional ethics review board guidelines. The inclusion criteria were:

(1) patients confirmed with lung adenocarcinoma by surgical resection;
(2) underwent routine contrast-enhanced CT of the entire thorax using the same CT machine with normalized reconstruction algorithm and thickness (1 mm), within two weeks of surgery; and
(3) immunohistochemical (IHC) examination of Ki-67 expression levels were detected within a week after surgery.

The exclusion criteria were:

(1) history of other malignancies or combined malignancies;
(2) CT imaging was reconstructed using different algorithms or thicknesses or if the reconstruction was performed on a different CT machine;
(3) underwent biopsy before CT examination; and
(4) neoadjuvant chemotherapy.

Based on the above-mentioned eligibility criteria, we included patients treated at our hospital between January 2017 and December 2017. A total of 95 patients (33 male and 62 female; mean age: 60.1 ± 12.5 years; age range, 33–74 years) met the requirements of our study. Clinical and pathologic information was collected from the hospital's electronic medical record system.

### 2.2 CT image acquisition

All patients underwent plain and vein phase contrast-enhanced CT of the entire thorax using a multi-detector CT system (Aquilion, Toshiba, Japan). The CT scan parameters were: tube voltage, 120 kV; tube current, 300 mA; pitch, 0.9; field of view, 180 × 180 mm; matrix, 512 × 512; and reconstructed slice thickness and slice increment of 0.5 mm. After plain CT, vein-phase contrast-enhanced scans were started at 35 s after the contrast media reached 100 HU. Contrast medium (300 mg/mL, Ioversol injection, Jiangsu Hengrui Pharmaceutical Co., Ltd.) was administered at 1.5 mL/kg of body weight and a rate of 2.5 mL/s. All images were exported in DICOM format for image feature extraction.

Two thoracic radiologists (HPW and SHZ, with 20 years’ experience in chest image interpretation) independently reviewed all the CT images on our PACS. A consensus was reached by discussion in case of disagreement. CT scans were reviewed as lung window images (window width = 1200 HU; window level = −600 HU) and mediastinal window images (window width = 350 HU; window level = 50 HU). Lesion size, spiculation, lobulation, and pleural indentation were evaluated as the morphological features of CT.

Lesion size was defined as the maximum diameter of the tumor on axial images. Spiculation was defined as the presence of linear strands that extend from the nodule or mass margin into the lung parenchyma without reaching the pleural surface. Lobulation was defined as a portion of the surface of a lesion that shows a shallow, wavy configuration, with the exception of the regions abutting the pleura. Pleural indentation was defined as a tumor with a linear structure that originates from the tumor and extends to the pleural surface.

### 2.3 Ki-67 assessment

The Ki-67 expression levels using typical tumor samples collected from the patients were routinely evaluated by experienced pathologists. IHC staining was performed in accordance with the manufacturer's protocol. Briefly, the formalin-fixed, paraffin-embedded tissue sections were cut into 4-μm sections, which were then dried and dewaxed in xylene, rinsed in graded ethanol, and rehydrated in double-distilled water. IHC staining was performed with the Ki-67 protein antibody (Beijing Zhongshan Jinqiao Biological Company, ZM-0166) at a dilution of 1:100. Cells with brown nuclei were considered as positive. After investigating the whole specimen, three regions with the highest density of positive cells were selected; 100 cells in each region were randomly counted under high magnification, and the Ki-67 index was calculated as the percentage of positive cells. The Ki-67 indices were calculated in the three regions and then averaged. The expression level of ki-67 was divided into five categories: ≤5%, 5%, 10%, 20%, and >30% of positive tumor cells.

### 2.4 Statistical analysis

Statistical analyses were performed using SPSS 17.0. A *P* value of < .05 was considered to be statistically significant. Differences were analyzed using the non-parametric test-K multiple independent sample test (Kruskal–Wallis test). Correlation analysis was performed using the Spearman correlation. Spearman correlation value of <0.40 indicates no correlation, while 0.40 to 0.70 values show moderate correlation, and >0.70 show high correlation.

## 3 Results

**Figure 1:**
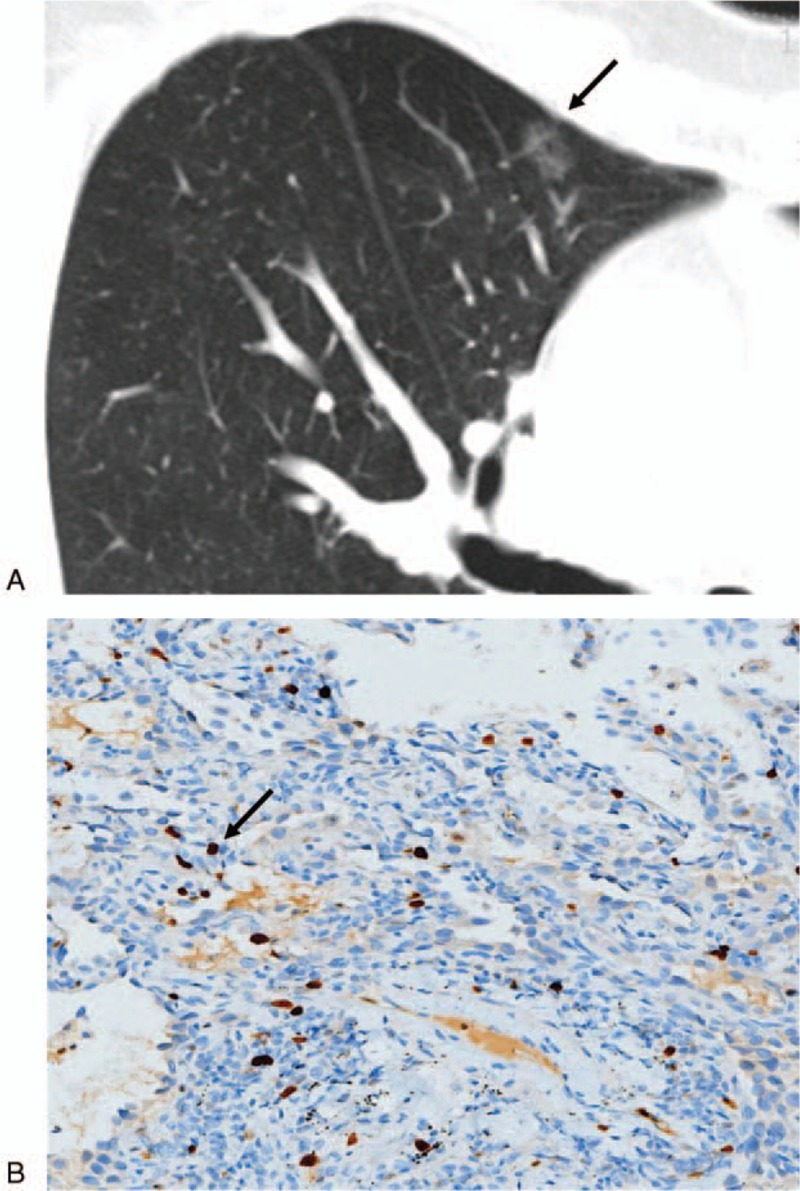
This nodule (arrow) was histopathologically confirmed as adenocarcinoma in situ (AIS). (A) Computed tomography image showing a ground-glass nodule. (B) The proliferative index was 5% in the glandular epithelium (Ki-67, × 200).

**Figure 2:**
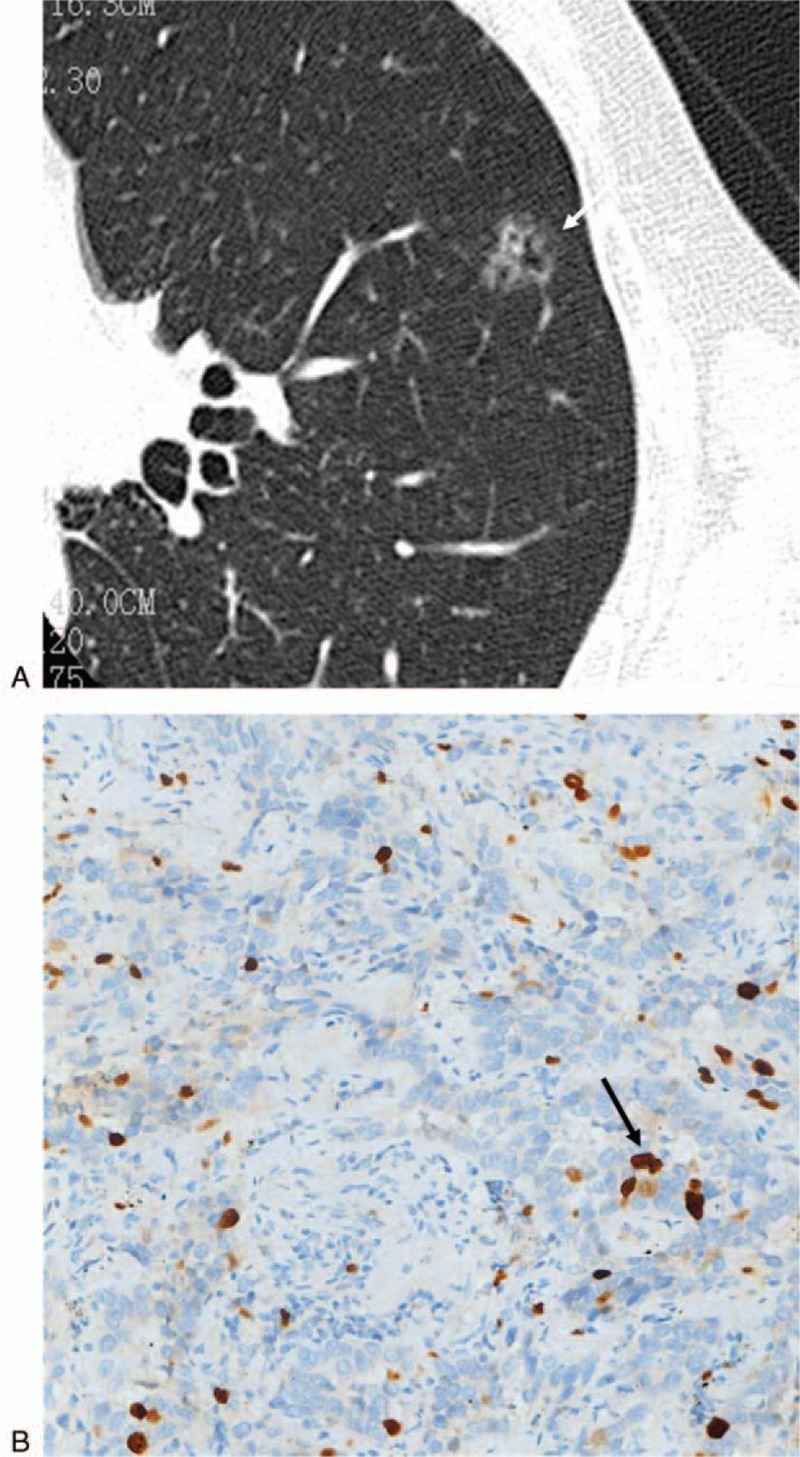
This nodule (arrow) was histopathologically confirmed as minimally-invasive adenocarcinoma (MIA). (A) Computed tomography image showing a part-solid nodule. (B) The proliferative index was 5% in the glandular epithelium (Ki-67, × 200).

**Figure 3:**
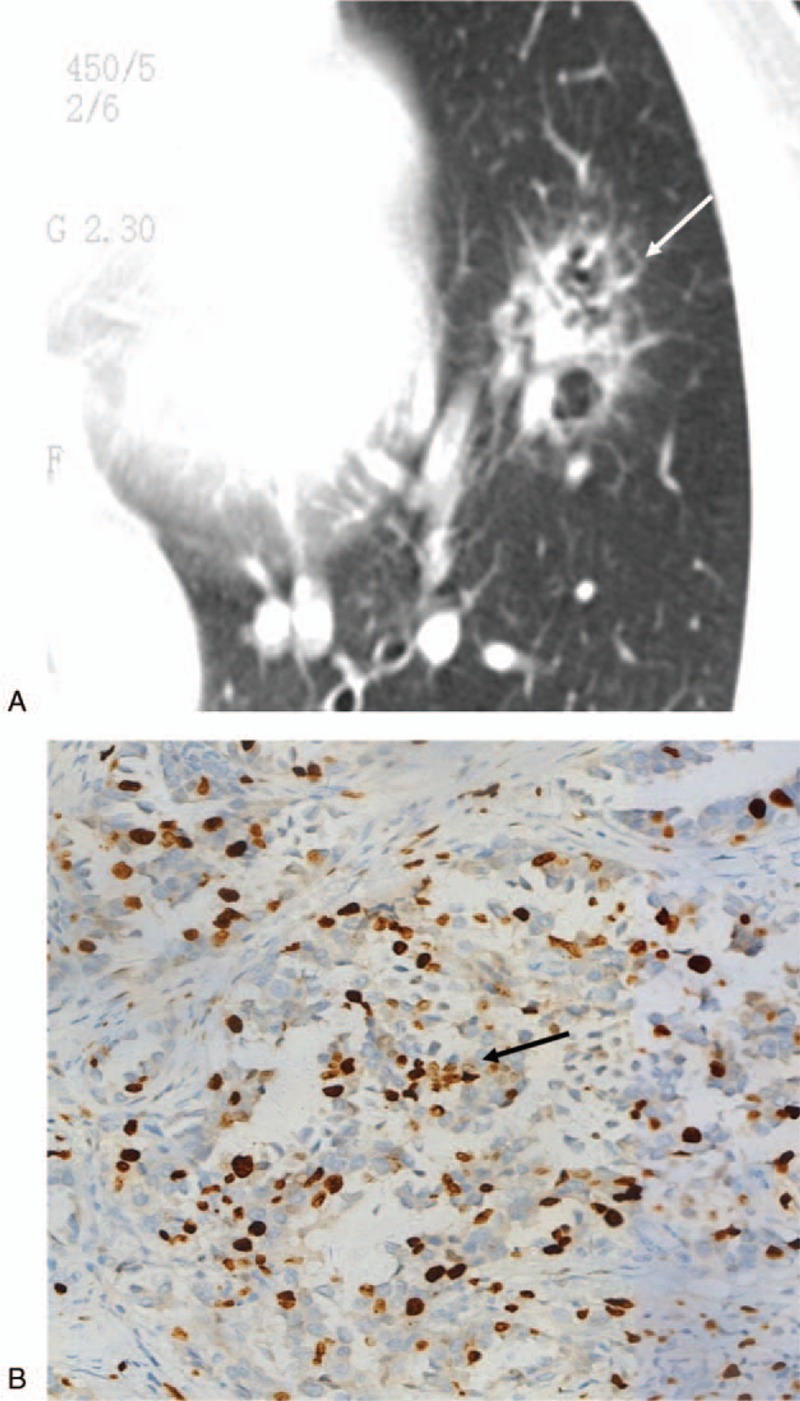
This nodule (arrow) was histopathologically confirmed as invasive adenocarcinoma (IAC). (A) Computed tomography image showing an irregular solid nodule. (B) The proliferative index was 20% in the glandular epithelium (Ki-67, × 200).

Pathological diagnoses based on multidisciplinary adenocarcinoma criteria were (Table 1): 36 patients had AIS, 32 had MIA, and 27 had IAC. Of the 36 patients with AIS, 58% (21/36) demonstrated Ki-67 expression level < 5% (Fig. 1) and 42% (15/36) Ki-67 expression level of 5%. Of the 32 patients with MIA, 44% (14/32) demonstrated Ki-67 expression level <5%, 41% (13/32) had 5% (Fig. 2), and 16% (5/32) had 10%. Of the 27 patients with IAC, 7% (2/27) had a Ki-67 expression level <5%, 15% (4/27) had 5%, 30% (8/27) had 10%, 26% had 20% (Fig. 3) and 22% (6/27) had ≥30%. The Kruskal–Wallis test showed that the lung adenocarcinoma subtype was associated with Ki-67 expression (χ^2^ = 41.142, *P* < .0001). The Spearman correlation coefficient was 0.615, showing that with the increase of the malignant degree from AIS to MIA to IAC, the level of Ki-67 expression also increased.

**Table 1:**
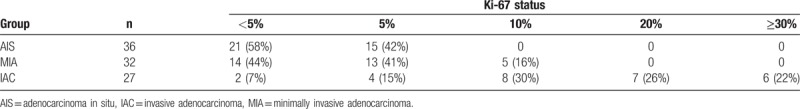
Correlations between Ki-67 staining and AIS, MIA, and IAC.

### 3.1 Correlations between nodule diameter/density and malignant signs and Ki-67 staining

The expression of Ki-67 and nodule diameter/density and malignant signs are shown in Table 2. The Spearman analyses showed that the expression of Ki-67 was positively related to the diameter, density, and lobulated sign of the lesion. The expression of Ki-67 showed no significant correlation with spiculations and pleural retraction.

**Table 2:**
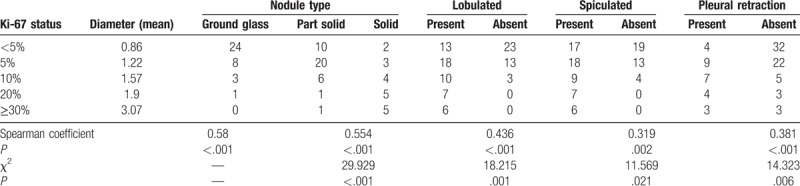
Correlation between the expression of Ki-67 and nodule diameter/density and malignant signs.

## 4 Discussion

Ki-67 is a cell proliferation marker and is mainly expressed during the active phases of the cell cycle, that is, G1, S, G2, and mitosis.^[6]^ A high cell proliferation rate is considered as a hallmark in cancer, and a high Ki-67 expression predicts poorer survival in patients with multiple myeloma, prostate cancer, and breast cancer.^[7–9]^ Studies revealed that glioma, bladder cancer, and anal cancer with high Ki-67 positivity are more aggressive and invasive.^[10–12]^ According to a meta-analysis, the Ki-67 labeling index has prognostic significance in patients with NSCLC, and a high index suggested a poor prognosis.^[13]^ Several studies have suggested an association of high metabolic PET parameters with high Ki-67 expression^[14–16]^ and worse survival in NSCLC patients.^[17–20]^ Therefore, the ability to accurately predict Ki-67 in preoperative imaging studies would help optimize therapeutic planning. Correlation between imaging features of tumors and cell proliferative activity as determined by Ki-67 expression level has been suggested in some studies on NSCLC.^[16,21,22]^

Currently, the relationship between NSCLC and Ki-67 mainly focused on the differences in the expression of Ki-67 between different pathological types. The relationship between different subtypes of lung adenocarcinoma and Ki-67 expression has not yet been reported. In particular, the correlation between the expression of Ki-67 and the different CT imaging features of pulmonary adenocarcinoma subtypes has not been clearly reported.

MSCT plays an important role in the diagnosis of lung cancer, and its imaging features can noninvasively predict tumor behavior. MSCT has shown promising value in lung cancer research in determining tumor viability or aggressiveness, response to chemotherapy and/or radiation, and genomic information. MSCT also has the ability to revolutionize the diagnosis, surveillance, and treatment planning of lung cancer patients, allowing for a personalized, noninvasive, and cost-effective management. To the best of our knowledge, no studies have examined the correlation between CT-based imaging features and Ki-67 status in lung cancer, and this is the first study to investigate this relationship. In the present study, the expression levels of different pathological subtypes of lung adenocarcinoma (AIS/MIA/IAC) and Ki-67 were analyzed. The expression degree of Ki-67 in the AIS group was mainly <5% (58%), and no expression of ≥10% was observed. In the MIA group, Ki-67 <5% (44%) was still the most frequent expression level, but Ki-67 expression was observed up to 20%. Only in the IAC group was Ki-67 expression ≥20% observed. Those results indicate that from AIS to MIA to IAC, the degree of malignancy increases, as shown by increasing Ki-67 expression. Lung adenocarcinoma with higher malignant degree will show more vigorous cell proliferation, faster tumor growth rate, and increased expression of Ki-67. Importantly, MSCT cannot replace biopsy and surgery to obtain specimens for a definitive diagnosis, but MSCT features could provide a noninvasive way for the early diagnosis of the lesions and improve patient management.

The correlation between the expression of Ki-67 in different lung adenocarcinoma subtypes and imaging characteristics was compared. The results showed that the degree of expression of Ki-67 was related to nodule diameter, density, and lobulations, and the correlation coefficients were 0.58, 0.554, and 0.436. It can be deduced that with the increase of the expression degree of Ki-67, the diameter of the nodules gradually increased, and the density was gradually increased from pure grinding glass to mixed grinding glass to solid nodules, which increased the rate of appearance of the lobulation signs. The reason may be that with the increase of Ki-67 expression, the cell growth and activity increase the number of tumor cells in the tumor, decrease the intercellular space, and increase the cell density, thereby gradually increasing the density on CT. The tumor will grow until all the space is occupied, and it becomes a solid nodule. Then, the tumor cells begin to expand outwards, and this occurs in a lobulated pattern due to the surrounding lobular septum and bronchial obstruction that offer different degrees of resistance to volume growth. On the other hand, there was no significant correlation with spiculations and pleural retraction, and the correlation coefficients were 0.319 and 0.381. The reason may be that the spiculations and pleural retractions are tumor-infiltrated stroma that results in cancer-associated fibroblasts (CAF). The spiculations and pleural retractions occur due to the contractive force of the CAFs, and they have little to do with cell proliferation. Therefore, the correlation between burr and pleural depression remained low.

This study is limited by its retrospective nature, with all the biases and limitations associated with such studies. The small sample size is also a limitation, preventing the analysis of the diagnostic power of the associations and correlations observed here. Additional studies are necessary to address those issues.

## 5 Conclusions

The MSCT signs of lung adenocarcinoma are related to the expression of Ki-67. Larger diameter of the mass, higher density, and more obvious lobulations were correlated with higher expression of Ki-67, while the malignant signs of spiculations and pleural retraction were not closely correlated with Ki-67. Therefore, without replacing biopsy and surgery, the noninvasive MSCT examination might indirectly reflect the proliferative activity of lung adenocarcinoma, which is of high value for early diagnosis, management, and prognosis determination.

## Author contributions

**Data curation:** Haiwei Zhou, Lin Qiu.

**Formal analysis:** Jingang Yan, Heping Wang, Zhaoyu Wang.

**Resources:** Hui He.

**Writing – original draft:** Jingang Yan, Heping Wang.
